# Fusing multidimensional hierarchical information into finer spatial landscape metrics

**DOI:** 10.1002/ece3.8206

**Published:** 2021-10-12

**Authors:** Gang Fu, Nengwen Xiao, Yue Qi, Wei Wang, Junsheng Li, Caiyun Zhao, Ming Cao, Juyi Xia

**Affiliations:** ^1^ College of Water Sciences Beijing Normal University Beijing China; ^2^ State Environmental Protection Key Laboratory of Regional Eco‐process and Function Assessment Chinese Research Academy of Environmental Sciences Beijing China; ^3^ State Key Laboratory of Environmental Criteria and Risk Assessment Chinese Research Academy of Environmental Sciences Beijing China; ^4^ School of Environment and Natural Resources Renmin University of China Beijing China

**Keywords:** hierarchy theory, information entropy, information volume, landscape ecology, spatial heterogeneity

## Abstract

One of the core issues of ecology is to understand the effects of landscape patterns on ecological processes. For this, we need to accurately capture changes in the fine landscape structures to avoid losing information about spatial heterogeneity. The landscape pattern indicators (LPIs) can characterize the spatial structures and give some information about landscape patterns. However, researches on LPIs had mainly focused on the horizontal structure of landscape patterns, while few studies addressed vertical relationships between the levels of hierarchical landscape structures. Thus, the ignorance of the vertical hierarchical relationships may cause serious biases and reduce LPIs' representational ability and accuracy. The hierarchy theory about the landscape pattern structures could notably reduce the loss of hierarchical information, and the information entropy could quantitatively describe the vertical status of landscape units. Therefore, we established a new multidimensional fusion method of LPIs based on hierarchy theory and information entropy. Here, we created a general fusion formula for commonly used simple LPIs based on two‐grade land use data (whose land use classification system contains two grades/levels) and derived 3 fusion landscape pattern indicators (FLIs) with a case study. The results show that the information about fine spatial structure is captured by the fusion method. The regions with the most differences between the FLIs and the traditional LPIs are those with the largest vertical structure such as the ecological ecotones, where vertical structure was ignored before. The FLIs have a finer spatial representational ability and accuracy, not only retaining the main trend information of first‐grade land use data, but also containing the internal detail information of second‐grade land use data. Capturing finer spatial information of landscape patterns should encourage the application of fusion method, which should be suitable for more LPIs or more dimensional data. And the increased accuracy of FLIs will improve ecological models that rely on finer spatial information.

## Graphical abstract

The ignorance of the vertical hierarchical relationships may cause serious biases reduced landscape metrics' representational ability and accuracy. Capturing finer spatial information of landscape patterns should encourage to understand the effects of landscape patterns on ecological processes.

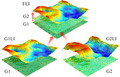

## 1 INTRODUCTION

The research of ecology increasingly requires multidimensional spatial data across broad extents, it is necessary to capture their fine spatial structures to avoid losing of information on spatial heterogeneity (Graham et al., 2019). The spatial heterogeneity is the basic concept of the landscape patterns, which is very important to understand the ecological processes. The landscape patterns result from complex relationships between multiple factors (Turner, 2005), and the study of landscape patterns is one of the core issues of landscape ecology (Frohn, 2006; O'Neill et al., 1988; Wang and Cumming, 2011; Wu, 2013). Landscape patterns and ecological processes influence and interact with each other, so the quantification of spatial heterogeneity is essential to clarify the relationships between ecological processes and spatial structure patterns. Therefore, the measurement, analysis, and interpretation of spatial patterns have attracted much attention in landscape ecology. One of the key issues of quantitative measurement and analysis of landscape patterns is the development and application of landscape pattern indicators (LPIs) (Wu, 2013), which can represent the key attributes of landscape patterns and provide important information for understanding the current status of landscape systems (Walz, 2015). LPIs are generally used to measure the spatial composition and configuration of landscape patches or characterize the overall features of the landscape range on a specific scale (Riitters et al., 1995). Meanwhile, LPIs also contain some information about the spatial structures, internal mechanisms, and action processes of landscape patches (Seppelt & Schröder, 2006). Because LPIs can reveal the potential links and ecological processes between organisms and substances within landscape patterns (Knoke et al., 2016), landscape indicators are also commonly used as quantitative measures to characterize the landscape pattern of ecosystems in studying species distribution, ecological processes, and habitat quality (Peng et al., 2020; Weisshuhn, 2019; Xie et al., 2020). Thus, enhancing the representational ability and accuracy of LPIs is essential for understanding, managing, and evaluating ecosystems, and carrying out ecological environmental protection management (Bundy et al., 2019).

There are diverse methods for landscape identification exist, due to the multidisciplinary nature of landscape concepts (Simensen et al., 2018). Among them, the gradient model (GM) (McGarigal et al., 2009) based on grid or net continuous data contains more information about spatial heterogeneity, and it is more meaningful to model landscape pattern and ecological process (Lausch et al., 2015). At present, most LPIs based on GM use the moving window approach to obtain rasterized metrics. Meanwhile, with the rapid development of remote sensing and geographic information systems, some tools such as Conefor (Saura & Torné, 2009) and Fragstats4.2 (McGarigal et al., 2012) provide convenient conditions for the application of LPIs. In the past decades, researchers have invented dozens LPIs, such as Shannon's diversity indicator (SHDI), path richness indicator (PR), number of patches indicator (NP), and other landscape heterogeneity indicators (Díaz‐Varela et al., 2016; Liu et al., 2014; Xia et al., 2020), to quantify landscape pattern. Due to the complexity of the landscape spatial model and the diversity of LPIs, it is difficult to uniformly measure and compare differential LPIs (Jia et al., 2019; Simensen et al., 2018; Tischendorf, 2001). However, the representational capability and accuracy of LPIs directly determines the application prospects and value of landscape indicators. Therefore, we can compare the performance of LPIs from their spatial heterogeneity and information.

**FIGURE 1:**
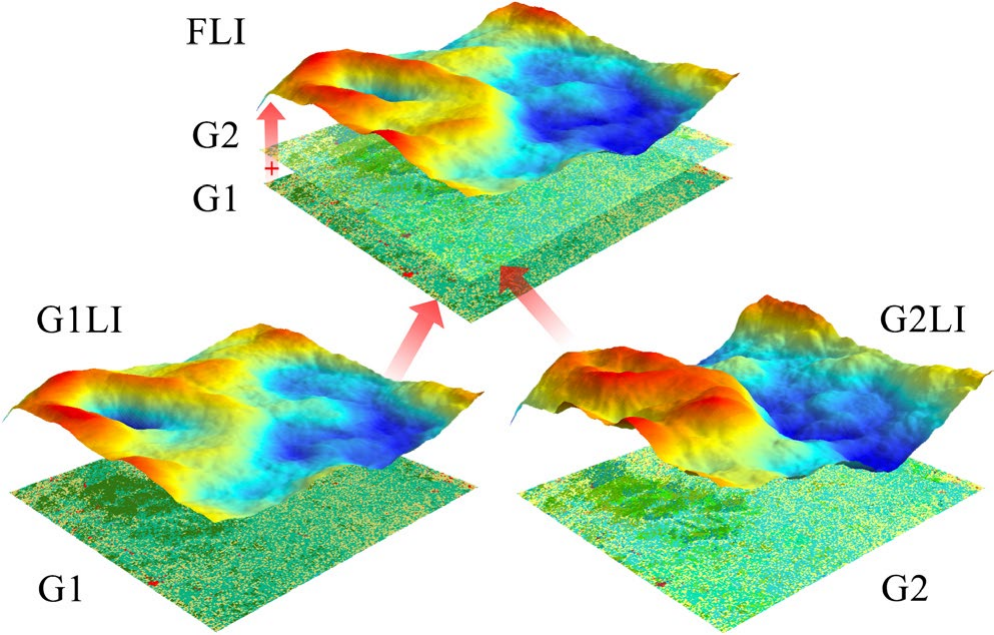
Schematic diagram of landscape indicators based on different land use data dimensions. G1 means the first‐grade land use and land cover (LULC), G2 means the second‐grade LULC, G1LI means the landscape indicator based on G1, G2LI means the landscape indicator based on G2, and FLI means the new fusion landscape pattern indicator. In practical applications, because G2LIs have some information redundancy, researchers generally only use G1LIs to study landscape pattern changes. If the changes in the landscape pattern only occur in the secondary structure, it is difficult to monitor the actual landscape changes from the original G1LIs. At this time, FLIs can accurately describe this subtle changes. This figure shows the differences between 3 indicators and their relationship to the grades of LULC

At present, the LPI measurement system contains dozens of characterization indicators with multifarious functions. However, most LPIs have some defects in the fine spatial structure, because they pay much attention to the horizontal spatial structure of the landscape, but ignore the vertical structure of the landscape, especially, failing to consider the vertical relationships of landscape patches between the different hierarchies. LPIs rely on land use and land cover (LULC) data, which are usually divided into multiple grades/levels according to the Land Cover Classification System (LCCS), and generally speaking, most LCCS (e.g., UNEP/FAO Land Cover Legend 1993) often includes two grades (Yang et al., 2017). Among them, the first‐grade LULC (G1) contains the main classes of land use, and the detailed classes of second‐grade LULC (G2) are divided from their corresponding G1. From a comprehensive comparison, we find that most LPIs have a certain degree of information consumption and loss, mainly because they fail to make full use of the current multilevel spatial data and the powerful computing power of computers, which makes it difficult to meet the needs of today's increasingly fine spatial LPIs. For example, the G1LI (landscape indicator based on G1 data) and the G2LI (landscape indicator based on ‐G2 data) only extract one level of land use data information in Figure 1. With the rapid development of remote sensing and computer technology, large multidimensional spatial remote data can be used to improve the accuracy of LPIs (Lausch et al., 2015). Therefore, a multidimensional data fusion method to generate a new fusion landscape pattern indicator (FLI), which contains more dimensional information with higher accuracy, is needed to make up for the systematic defects ignoring the vertical relationship of the landscape pattern.

The systematic perspective in system ecology proposes a hierarchy theory (Jorgensen et al., 2016), which implies that both vertical relationships and horizontal connections of their interactions come into play should be considered (Jørgensen & Nielsen, 2015). Many landscape ecologists have imported hierarchical theory from systems ecology into the study of landscape ecology (Wu & David, 2002); that is, landscape patterns can be perceived as nested spatial hierarchies. Therefore, the horizontal structure of the landscape refers to the relationships between the landscape units at the same grade/level, and the vertical structure of the landscape refers to the relationships between the landscape units at the upper and lower levels. And the constraint envelope concept within hierarchy theory has also helped to enhance the understanding of landscape patterns (Gustafson, 2019). However, for a long time, the constraint mechanism of the upper and lower subsystems based on hierarchy theory (Rose et al., 2017) was not effectively applied in the study of LPIs. Furthermore, information entropy theory offers a reliable framework for the study of landscape patterns (Nowosad & Stepinski, 2019), which could quantitatively describe the status according to the entropy distribution characteristics of landscape units (Antrop, 1998). Here, we integrated the discrete mosaic patches of two‐grade LULC data on the vertical and horizontal gradients, with the aim of (1) establishing a hierarchical framework of LPIs based on hierarchy theory and using information entropy theory to quantitatively describe those hierarchical relationships, (2) generating a series of FLIs to immerse more information in both horizontal and vertical structures, and (3) verifying the advantages of FLIs through the information volume based on the optimal encoding method. We have written the Python codes based on the fusion framework to easily implement 3 FLIs calculations and supplied two tools for quantitatively and uniformly measuring the information volume of spatial indicators.

## 2 THEORY

### 2.1 The modeling framework

**FIGURE 2:**
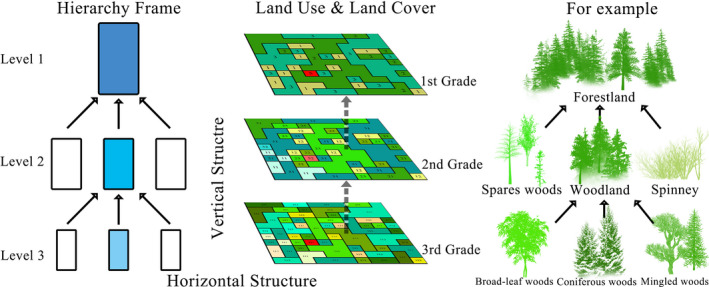
Illustration of the major concepts in the hierarchy theory of landscape. The figure on the left shows the multilevel structure of the hierarchy frame, while the figure on the right shows the hierarchical relationship of the forestland by taking the forest as an example. The numbers on the central part of the figure are the classification codes for land use and land cover (modified from Wu & David, 2002)

Hierarchical theory can help to understand landscape patterns (O'Neill et al., 1992) with spatially nested hierarchical structures and can determine the critical range of the target layer by analyzing higher‐level constraints and lower‐level constraints (Gustafson, 2019). According to hierarchy theory, complex landscape components include both a multilevel vertical structure and a horizontal heterogeneous structure that consists of patch classes (Wu & David, 2002). Among them, the relationship between two adjacent levels is asymmetric, which means that the upper level exerts constraints to the lower level, whereas the lower level provides initiating conditions to the upper level. Meanwhile, the neutral theory (O'Neill et al., 1992) considers that the relationship between patch classes at the same level is relatively symmetric, and the interactions among subunits within the same landscape patch class are stronger and more frequent than those between classes in Figure 2.

Various types of entropy have been imported into landscape ecology, such as information entropy (Díaz‐Varela et al., 2016; Rocchini et al., 2013), spatial entropy (Wang & Zhao, 2018), and thermodynamic entropy (Gao and Li, 2019a, 2019b), and now they are widely used by ecologists. Generally speaking, the essence of entropy is the intrinsic chaos and uncertainty of a system. In this study, when the landscape only contains one patch, its entropy is the lowest. As patch classes increase, the value of entropy becomes larger and larger. When the landscape includes all patch classes, which are maximum uniform distribution, its entropy is the largest. The hierarchical information association based on the subordinated relationships between LULC categories or landscape patch classes in vertical grades can be established by the information entropy theory to fuse multilevel data for LPIs. Due to the hierarchical levels and the subordination relationships of multi‐grade LULC data, we used information entropy to quantitatively describe the hierarchical association between upper and lower LULC classes with subordination relations; that is, we described the distribution of secondary landscape patches within the superior landscape patches based on information entropy.

There are many classification systems for LULC, including both one‐grade classification and multi‐grade classification. However, even if there is a large amount of multi‐grade LULC data, only one‐grade LULC data (e.g., G1 or G2) are used to calculate LPIs, which may lead a loss of spatial information. The most commonly used primary classification system is generally divided into several categories, such as cropland, forestland, grassland, wetland, constructive land, and unutilized land, which are also the core landscape patch classes of most LPIs. In this research, we established a framework to generate and demonstrate the fusion of LPIs as follows:

1. Clarifying the hierarchy constraint relationships of multidimensional LULC data between the upper and lower categories.
2. Describing the configuration association of lower landscape patch classes within upper ones by information entropy theory, with the first‐grade LULC as the target layer.
3. Fusing multidimensional data by using the information entropy weight of hierarchy constraint relationships and visualizing FLIs by using the moving window method under the GM.
4. Calculating the information volume of the FLIs and traditional LPIs to assess the accuracy and presentational ability of FLIs.

### 2.2 The two‐dimensional LULC fusion algorithm

#### 2.2.1 The fusion formulae of SHDI, PR, and NP

Shannon (1948) proposed the principle and method of information entropy from the perspective of probability theory and mathematical statistics and defined the information entropy as the reduction of the degree of random uncertainty. In the study of landscape patterns, the information entropy can measure the degree of disorder of the system (Brunsell, 2010), which can be used to quantitatively describe the states of landscape patch classes between different levels with subordinate relationships (Wang & Zhao, 2019). Therefore, we used the conditional entropy of the nonrandom discrete variable and maximum entropy to express the uncertainty of second‐grade landscape patches within the first‐grade landscape patches, according to the subordinate relationship within the classification of LULC data. Thus, the FLI formulae could be constructed by hierarchy theory and information entropy.

In this study, we used two‐grade LULC data as an example with which to show the calculation process of the fusion landscape indicators with first‐grade LULC (G1) as the target layer. First, we divided the patch classes of two‐grade LULC data into sets X and Y, where X is the set of all patch classes in raster data G1, and Y is the set of all patch classes in second‐grade LULC (G2). *x_i_* is an element of the X set, *x_i_* ∈ X, and *y_ij_* is an element of the Y set, *y_ij_* ∈ Y. *Y_i_* is a set of *y_ij_* subordinated to upper‐level *x_i_*, *y_ij_* ∈ *Y_i_*, *Y_i_* ⊆ Y. And the relationship between *x_i_*, *Y_i_*, and *y_ij_* is illuminated in Figure S1.

The information entropy *H*(*X*) based on discrete random variables is calculated by

(1) $$H\left(X\right)=‐\sum_{x_{i}\in X}p\left(x_{i}\right)*\ln\left(p\left(x_{i}\right)\right)$$

where *p* (*x_i_*) is the proportion of *x_i_* in the target layer.

According to the conditional entropy of the formula and membership relationship, *H*(*Y|X*) is the entropy of the Y set conditioned on the X set, calculated by

(2) $$H\left(Y|X\right)=‐\sum_{x_{i}\in X,}p\left(x_{i}\right)*\sum_{y_{\mathrm{ij}}\in Y_{i}}p\left(y_{\mathrm{ij}}|x_{i}\right)*\ln\left(p\left(y_{\mathrm{ij}}|x_{i}\right)\right)$$

According to formula (2), $H_{i}^{\prime }$ is the entropy of the Y set conditioned on *x_i_*, calculated by

(3) $$H_{i}^{\prime }=‐p\left(x_{i}\right)*\sum_{y_{\mathrm{ij}}\in Y_{i}}p\left(y_{\mathrm{ij}}|x_{i}\right)*ln\left(p\left(y_{\mathrm{ij}}|x_{i}\right)\right)$$

(4) $$p\left(y_{\mathrm{ij}}|x_{i}\right)=\frac{p\left(y_{\mathrm{ij}}\right)}{p\left(x_{i}\right)}$$

where *p* (*y_ij_*) is the proportion of *y_ij_* in the *x_i_*.

When the landscape patches are completely randomly distributed in the area, that is, the patches are completely uniformly distributed, and then, the information entropy is the largest (Wang & Zhao, 2019), so the maximum entropy of *Y_i_* is ln (*n_i_*), where *n_i_* is the total number of elements in set *Y_i_*. In the hierarchical system, the upper‐grade patch classes constrain the lower‐grade patch classes, so we used conditional entropy (Nowosad & Stepinski, 2019) and maximum entropy to evaluate the entropy uncertainty (Moreno & Jesus Lopez, 2013) of class level. According to formula (3) and maximum entropy, the weight factor *w_i_* of *Y_i_* set to *x_i_* is calculated by

(5) $$w_{i}=\frac{H_{i}^{\prime }}{\ln(n_{i})}$$

In this study, we used 3 LPIs including the SHDI, PR, and NP to integrate the corresponding FLIs. An overview of the 3 employed LPI formulae, which are commonly used classic simple indicators in the study of landscapes, is provided in Table S1. For the 3 LPIs, their general formula *LI_X_* can be expressed as the aggregation of the corresponding component vector *LI_i_*, calculated by

(6) $$LI_{X}=\sum_{i}LI_{i}$$

where *LI_i_* is the indicator value of *x_i_* on the landscape patch class level or repeated part.

In the fusion process of two‐grade landscape patterns, we took the G1 as the core and fused the spatial information of G2 into its corresponding landscape patch classes of G2. Therefore, the fusion formula *FLI_X_* is calculated by

(7) $$FLI_{X}=LI_{X}+\sum_{i}w_{i}*LI_{i}=\sum_{i}LI_{i}+\sum_{i}w_{i}*LI_{i}=\sum_{i}\left(1+w_{i}\right)*LI_{i}$$

According to formulae (3), (5), (6), and (7), the general fusion formula *FLI_X_* is calculated by

(8) $$FLI_{X}=\sum_{i}\left(1‐\frac{\left(p\left(x_{i}\right)*\sum_{y_{\mathrm{ij}}\in Y_{i}}p\left(y_{\mathrm{ij}}|x_{i}\right)*\ln\left(p\left(y_{\mathrm{ij}}|x_{i}\right)\right)\right)}{\ln\left(n_{i}\right)}\right)*LI_{i}$$

In this paper, according to the formula characteristics of SHDI, PR, and NP, we obtained their FLIs' calculation formulae (Table S2).

#### 2.2.2 Visualized demonstration of FLIs by the moving window approach

**FIGURE 3:**
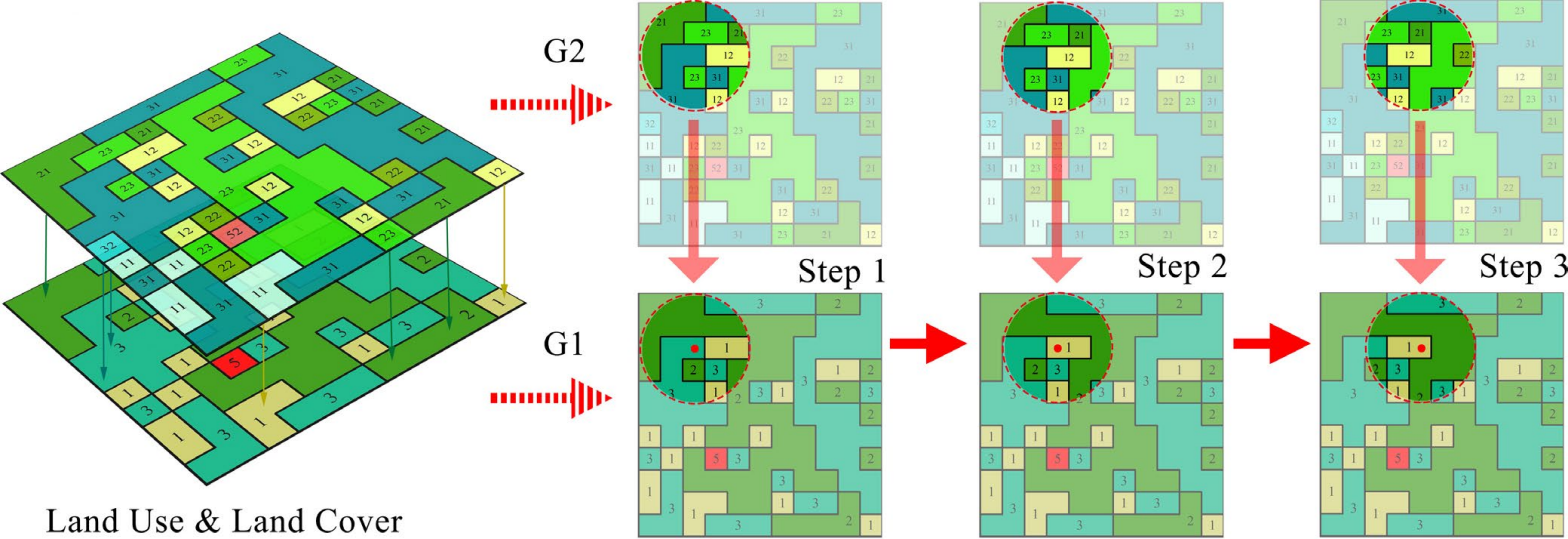
Schematic diagram of the calculation process of fusion indicators. G1 means the first‐grade land use and land cover (LULC), G2 means the second‐grade LULC, the red dotted frames are the circular moving window, the red dots are the center points of the moving window, and the numbers on landscape patches are the corresponding land use categories codes, which are detailed in classification Table S3

The moving window approach moves a window of a certain shape and size across a raster map and assigns the value of a landscape metric calculated within that window to the cell over which the window is centered (Graham et al., 2019; Lausch et al., 2015). The continuous gradient grid set of LPIs can be obtained by using the moving window approach, which is also a common method in the GM framework to visualize the demonstration of FLIs. First, according to the calculation process of the moving window approach in Figure 3, we fused the G2 data in the window into G1. We calculated the landscape indicator value in the window and then assigned the value to the center point. Second, we continued to move the windows one by one until the entire area was calculated to obtain the final FLIs. The entire process was calculated in Python 2.7 with the help of Fragstas4.2 software and ArcGIS 10.5. For more details, the complete code has been provided as a Python supplemental archive.

## 3 MATERIALS AND METHODS

### 3.1 Data and study area

**FIGURE 4:**
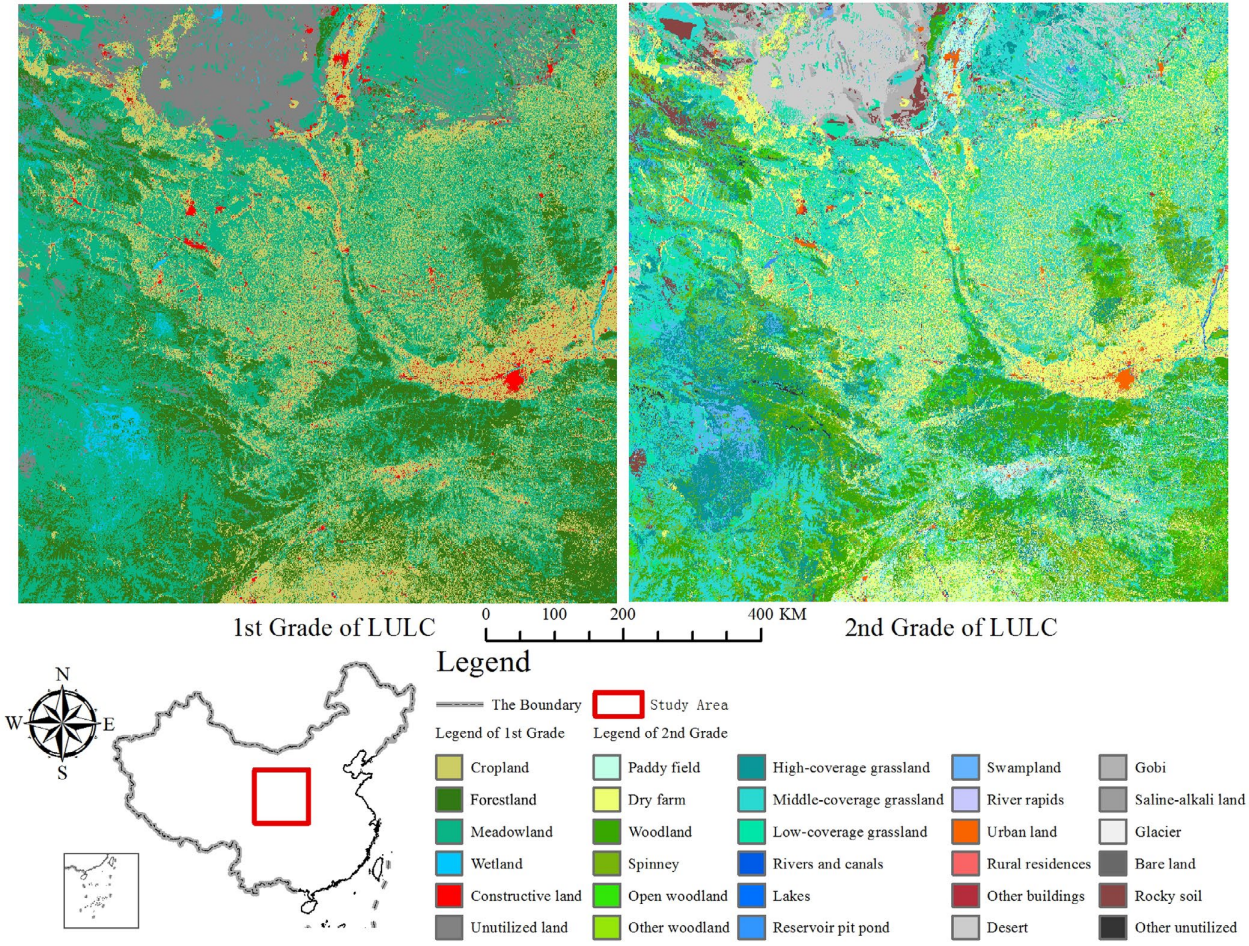
Land use and land cover (LULC) map and location map of the study area. These LULC data include 6 first‐grade categories and 24 second‐grade categories

The study area is located in central China, with a typical inland climate. The study area extends over 31°21′N to 39°9′N and 100°41′E to 110°57′E, with a total geographic area of 7.58 × 10^5^ km^2^. The area is bordered by the Guanzhong Plain in the east, desert and grassland in the north, Qinling Mountain in the south, and Qinghai–Tibet Plateau in the west. The landscape pattern classes in this area are various and abundant, including almost all continental landscape categories, and it is one of the regions with the richest landscape diversity in the word. The LULC dataset of 2018 (spatial resolution is 1 km × 1 km) was provided by the Data Center for Resources and Environmental Sciences, Chinese Academy of Sciences (RESDC) (http://www.resdc.cn). The LULC dataset was divided into two grades, including 6 first‐grade categories and 24 second‐grade categories (Figure 4 and Table S3).

### 3.2 Quantitative analysis of information volume

LPIs must fulfill the function of information and take advantage of various measurable data to provide a highly objective picture of the landscapes (Walz, 2015). It is very important to control information loss and to quantitative analysis of the information contained in the LPIs, which can effectively measure their representational ability and accuracy (Gann, 2019; Liang et al., 2021; Stoy et al., 2009; Wei et al., 2017). To compare the spatial heterogeneity of FLIs, G1LIs, and G2LIs, we calculated the information volume of those indicators based on information theory and conducted quantitative analysis from the perspective of information volume. The Huffman tree method is an encoding method that constructs an optimal binary tree with the shortest weighted path length (Hermassi et al., 2010). And this approach (Huffman, 1952) was first used in the field of communication and is an information entropy coding algorithm capable of lossless information compression. When accurately compare and measure the amount of information contained in each landscape indicator, we selected the first 4 decimal places of the indicators' normalized value using the dichotomy weighting method, which account for >90% of the total information volume. The Python codes and more details have been provided on https://github.com/ecofg/FLIs_fusion‐metrics.

## 4 RESULT

### 4.1 The FLI datasets and the spatial correlation between FLIs and LPIs

**FIGURE 5:**
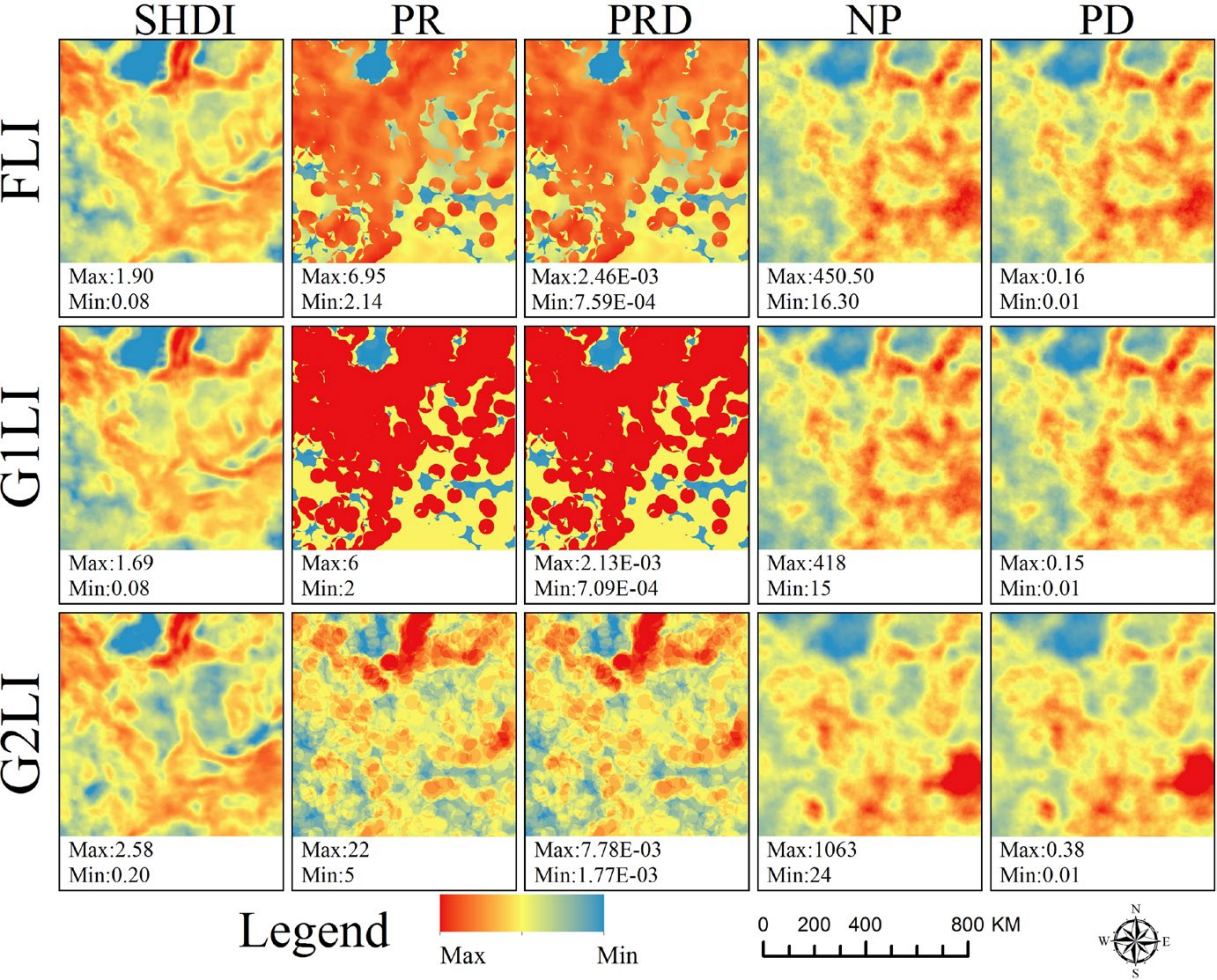
The datasets of FLIs (FLI‐SHDI, FLI‐PR, and FLI‐NP) at a series of RMW from 10 to 50 km. RMW means the radius of moving widows

We applied the fusion method to the study area and got the FLIs datasets (Figure 5) of fusion Shannon's diversity indicator (FLI‐SHDI), fusion path richness indicator (FLI‐PR), and fusion number of patch indicator (FLI‐NP) on different moving window scales (the radius of moving windows is 10, 20, 30, 40, and 50 km). The datasets showed that the moving window scale significantly affected the spatial features of FLIs.

The comparison of datasets between FLIs and traditional LPIs showed that the FLIs had a high correlation with G1LIs, which were greater than 0.90 (Figure S1). Meanwhile, the spatial correlation coefficients between FLIs and G2LIs were obviously higher than those coefficients between G1LIs and G2LIs. It indicated that the spatial distribution characteristics of the FLIs were mainly inherited the spatial characters of the G1LIs, and retained the spatial information of G2LIs. The results obtained from the preliminary datasets and correlation heat map showed that the FLIs included spatial information about the G1LIs and G2LIs.

### 4.2 The importance of FLIs in spatial distribution

**FIGURE 6:**
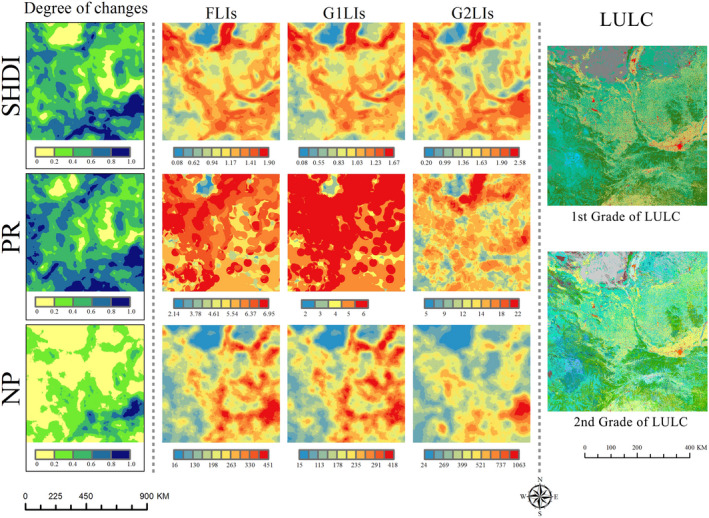
The spatial differences between FLIs and LPIs. The moving window scale is 30 km, and the degree of changes is calculated by the absolute value of difference between FLIs and G1LIs

The FLIs have some new fine spatial characteristics, in order to further clarify the importance of FLIs in more detail, we took FLIs and LPIs on the window scale of 30 km as an example to conduct a spatial comparison and analysis. The FLIs succeeded the main distribution characteristics of the G1LIs and performed additional refinement spatial structure characteristics of the G2LIs, which were not present in the traditional LPIs (Figure 6). And the FLIs had better numerical continuous gradient changes, which could reflect spatial heterogeneity with more accuracy.

We defined the degree of changes as the difference between FLIs and G1LIs and set its value range to [0,1] (Figure 6). It showed that the spatial distribution of FLIs and G1LIs had both similarity and heterogeneity. However, those areas with larger differences (the degree of changes > 0.6) were showed that FLIs had significant advantages in fine spatial structures. Compared with traditional G1LIs, the FLIs integrated more hierarchical information from G2LIs with higher spatial heterogeneity; that is, the new FLIs made up for the fine spatial information that G1LIs missed on G2LIs. Therefore, the FLIs have a better spatial representational ability and accuracy, especially comprehensively displaying new spatial structure information. For example, the 3 FLIs had their own advantages in fine spatial representation, but the dominant regions of the 3 FLIs were mainly located in different ecotones. We found that regions with the most differences between the FLIs and the traditional LPIs were those with the largest vertical structure such as ecological ecotones, where their vertical structures were ignored before. Taking the forestland–grassland ecotones in this study as an example, the G1LIs ignore the ecological impact of land change within the second‐grade categories of forestland, such as the G1LIs cannot identify the conversion of woodland to spinney/ shrubland. Meanwhile, G2LIs are difficult to distinguish the differences between second‐grade categories across first‐grade groups. From the perspective of general ecological effects, the land use changes in woodland to spinney/shrubland are weaker than woodland to grassland, and FLIs can quantify those processes, but G2LIs cannot effectively distinguish their ecological differences. In general, the FLIs retain the primary trend information of the G1LIs and contain the finer spatial information in internal details. Therefore, the FLIs have more dimensional information, which can make up for the defects of the vertical weight of G1LIs and G2LIs, and thus have better accuracy.

### 4.3 The validation in information volume

The information volume based on the Huffman tree optimal coding method revealed that the FLIs had more steady information, and it implied that the more information, the greater spatial heterogeneity. It showed that the total information volume of FLIs was at a very high level; in particular, the information volume of FLI‐PR and FLI‐NP was more than twice that of G1LIs on different window scales (Figure S3).

For more precise comparison, we calculated the spatial information volume with a window scale of 30 km (Figure S4). Regarding the spatial distribution of information volume, the information volume of FLIs was also better than that of G1LIs and G2LIs, showing better representational ability. The 3 FLIs could reflect dissimilar landscape pattern information and had obvious spatial heterogeneity in the spatial distribution of information volume. The spatial matching Pearson correlation coefficients revealed that FLIs and LPIs had a degree of significant spatial correlation with their corresponding information volume, but their relevant degrees were relatively low (Table S4). It implied that there were low relevant relationships between the spatial information volume and corresponding raster indicators, and the spatial information volume might be more related to their spatial heterogeneity than the indicators themselves. Therefore, the greater the spatial information volume of raster indicators, the greater their spatial heterogeneity.

**FIGURE 7:**
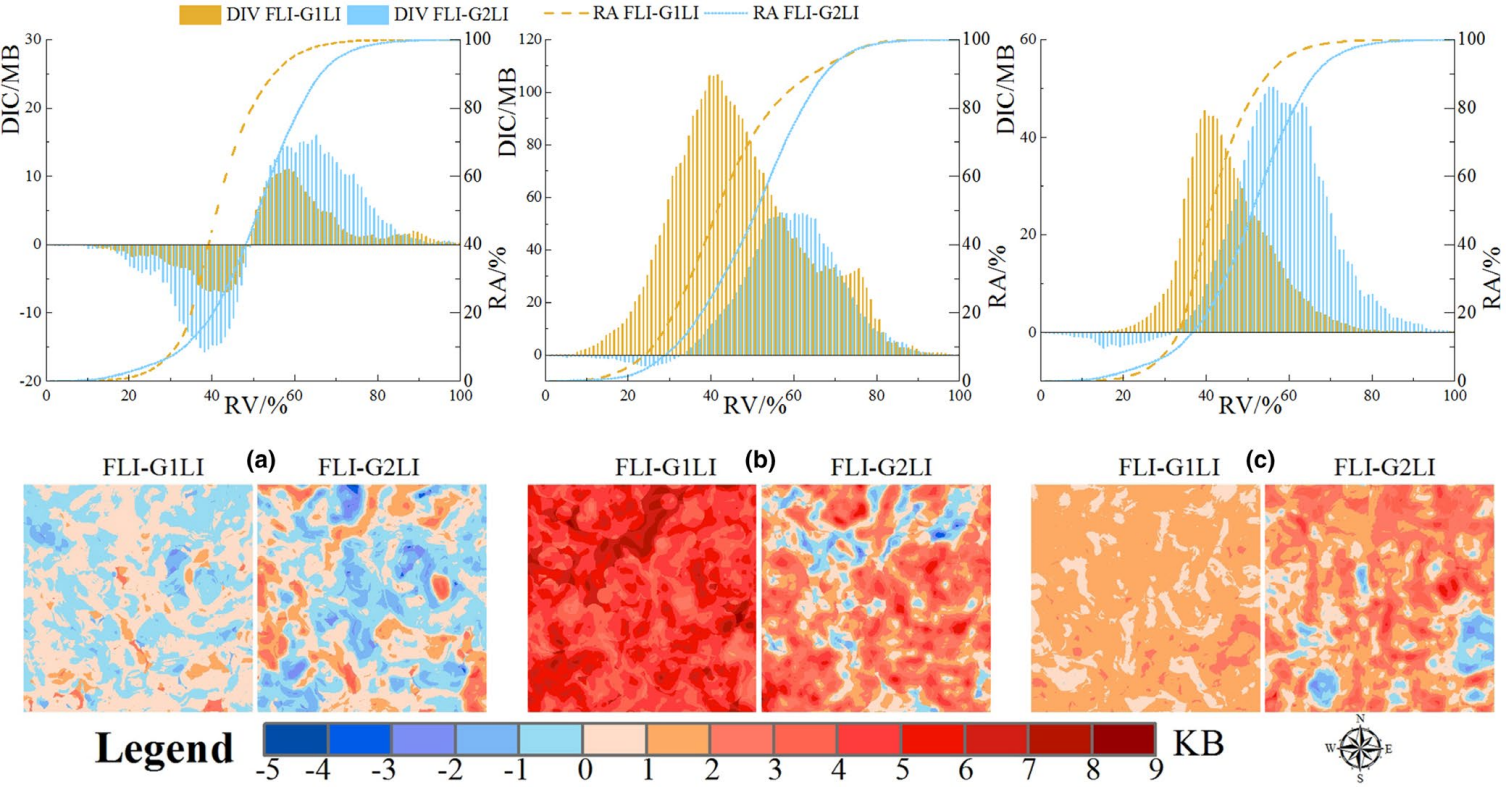
The difference in spatial information volume between FLIs and LPIs. (a) SHDI, (b) PR, (c) NP. FLI‐G1LI means the difference in information volume between FLIs and G1LIs, FLI‐G2LI means the difference in information volume between FLIs and G2LIs, DIV means the differences in information volume between FLIs and LPIs, RV means the ratio of normalized difference values between FLIs and LPIs, RA means the area ratio, KB is 2^10^ bits, MB is 2^20^ bits

We quantitatively compared the differences between the FLIs and LPIs from the spatial distributions of the information volume, and this comparison revealed that the FLIs not only had advantages in total information volume, but also had obvious advantages in spatial distribution, with the information volume of 3 FLIs being higher than that of 3 LPIs in 84.02% area (Figure 7). Meanwhile, we found that the FLIs' information mainly came from the G1LIs', because the regression coefficients in the equations illuminated that the information volumes of the G1LIs played a more significant role than that of G2LIs in explaining the information volumes of the FLIs (Table S5). It could be shown from both the spatial distributions of FLIs (Figure 6) and their spatial information volume (Figure S3 and Figure 7) that the accuracy of multidimensional FLIs was higher than that of single‐dimensional LPIs. Therefore, the FLIs have more dimensional information and finer spatial characteristics.

## 5 DISCUSSION

We proposed a fusion modeling framework for landscape metrics using hierarchy theory and information entropy, which was fundamentally different from the traditional LPIs. And the results showed that the new FLIs had more landscape information in both vertical two‐grade land use data and horizontal patterns between landscape patches. The FLIs contained higher spatial heterogeneity and finer spatial structures, not only retaining the main trend information of the G1LIs, but also containing the G2LIs' information in internal details. Because the G1LIs with the spatial structure characteristics of first‐grade categories could express the main information of landscapes, but they could not show more detailed internal features of landscape patch classes. Meanwhile, the G2LIs with the spatial structure characteristics of second‐grade categories could give refined exhibitions of the second‐level landscape patch classes, but the information of the G2LIs was redundant, due to the lack of weighting coefficient for landscape path classes. However, FLIs not only had the main trend characteristics of G1LIs, but also improved the internal variability of the landscape patch classes in the first grade. Therefore, the FLIs could simultaneously reflect the characteristics of their horizontal and vertical structures, so the FLIs had better application value on fine spatial structures.

### 5.1 Applicability of the fusion method

Landscape indicators play a principal role in the measurement of landscape patterns, and their accuracy determines their application (Fan & Myint, 2014). There are various LPIs in landscape ecology, whose measurable accuracy and representational ability also have their own advantages and disadvantages (Babí Almenar et al., 2019). The fusion of different spatial thematic resolution datasets is a key issue in related scientific research fields (Chen et al., 2019). For example, LULC datasets sometimes need to downscale or upscale to another spatial resolution to meet the requirements of corresponding researches. In this transformation, some spatial information may be lost, while the multidimensional algorithm can reduce information loss in aggregating categorical data (Gann, 2019). However, in the past, some LPIs generally ignored the multilevel vertical structure of the landscape pattern, which led to the underutilization of multilevel data and the loss of information. The core of this fusion method is to build a reliable multilevel connection between hierarchy patches. Moreover, establishing a dependable relationship between spatiotemporal multisource and multiscale data can further improve the utilization efficiency of those data and the accuracy of landscape metrics (Buyantuyev et al., 2010).

### 5.2 The influencing factors of FLIs

There are many factors affecting the accuracy of FLIs, including the scale effect and the classification system (Buyantuyev & Wu, 2007; Liu et al., 2013). (1) The scale issue is one of the core of LPIs, which is an essentially inherent law in the hierarchical system of nature (Cheng et al., 2008). The structure and functions of landscape patterns are scale‐dependent, and those studies are always carried out on a certain premise scale (Liu et al., 2011). Thus, the influence of the scale effect should be paid attention in future studies. (2) The classification system of LULC determines the classes of heterogeneous landscape patches, which directly affects the composition of landscape pattern and spatial structures (Dale & Kline, 2013), and the classification system is regarded as the primary issue in constructing the hierarchical relationship of the FLIs. (3) The FLIs are also affected by computer performance. The fusion method improves the utilization efficiency of multidimensional LULC data with more calculation, but the performance of professional computer can fully accommodate the additional calculation.

### 5.3 The measurement of information

In the past, the information measurement methods of LPIs usually used Shannon information entropy (Karasov et al., 2020), principal component analysis, or stepwise linear regression models (Wei et al., 2017); although these methods have the advantages of simplicity and speed in measuring the amount of information, they are not accurate enough and difficult to uniformly measure the spatial heterogeneity across LPIs. The Huffman tree optimal coding method (Srikanth & Meher, 2013) is the most efficient binary encoding method from the perspective of information theory. We first imported this method into the field of landscape metrics, which quantitatively calculated the information volume of the LPIs. Moreover, in the process of information statistics, we only counted the binary code lengths of all numerical values of raster data, while the spatial topological relationship of landscape patterns was not considered. LPIs contain information on the internal mechanisms of landscape patterns (Seppelt & Schröder, 2006), the larger the information volume of those indicators, the more ecological process information is contained, which also shows that the accuracy of those indicators is higher (Shao & Wu, 2008). Although the detailed relationships between LPIs' information volume and the representational ability require further study, it is still an effective method to measure the information content and fine spatial structure of LPIs based on information theory.

We provided a general fusion formula for two‐grade LULC data and applied this method to the fusion of SHDI, PR, and NP, which was an effective way to solve the fusion issues of multidimensional landscape data. A suggestion for future research is related to making full use of the huge spatial data and computer computing performance to greatly improve the representational ability of landscape indicators. For future study, we look forward to applying hierarchical theory and information entropy to achieve the fusion of multidimensional and multiscale landscape data for more LPIs. And we believe that the general fusion framework may be suitable for more LPIs. Furthermore, the fusion method can be improved for 3 or more dimension (or grades/ levels) LULC data. We take an improved fusion method of three‐grade land use data as an example. First, we need to set the second‐grade layer as a core layer and then fuse the third‐grade landscape patch classes into their corresponding categories of second‐grade layer, respectively. Second, after re‐targeting the first‐grade layer as a new core layer, we once again merge the fusion second‐grade category data in the first step into the first‐grade categories, respectively. Finally, according to the landscape pattern indicator's formula, the FLIs of three‐grade LULC can be calculated step by step. However, it should be noted that as the data's dimensions (or levels/ grades) increase, the calculation process becomes more and more complicated.

## CONFLICT OF INTEREST

None declared.

## AUTHOR CONTRIBUTIONS

**Gang Fu:** Conceptualization (equal); Methodology (lead); Software (equal); Writing‐original draft (lead); Writing‐review & editing (equal). **Nengwen Xiao:** Funding acquisition (lead); Software (equal); Validation (equal). **Yue Qi:** Data curation (lead); Formal analysis (equal); Resources (equal). **Wei Wang:** Conceptualization (lead); Project administration (equal); Writing‐original draft (equal); Writing‐review & editing (lead). **Junsheng Li:** Conceptualization (equal); Funding acquisition (equal); Methodology (equal); Project administration (lead); Supervision (equal); Writing‐review & editing (equal). **Caiyun Zhao:** Formal analysis (equal); Funding acquisition (equal); Software (equal); Validation (equal). **Ming Cao:** Data curation (equal); Formal analysis (equal); Resources (equal). **Juyi Xia:** Data curation (equal); Formal analysis (equal); Resources (equal); Software (equal).

## Supporting information

Supplementary Material

Fig S1

Fig S2

Fig S3

Fig S4

Fig S5

## Data Availability

The Python codes, the datasets, and the supplementary material documentation are hosted at https://github.com/ecofg/FLIs_fusion‐metrics.
