# Supplementary material for: Fusing multidimensional hierarchical information into finer spatial landscape metrics
Source: Ecol Evol. 2021 Oct 12;11(21):15225–36. doi: 10.1002/ece3.8206 (PMC8571621; doi:10.1002/ece3.8206)
Supplement: Supplementary file 1 — Supplementary Material [file ECE3-11-15225-s005.docx]

### Supplementary Material

**TABLE**

**TABLE** **S1** The 3 employed LPIs

| Names | Codes | Formulae | Description |
| --- | --- | --- | --- |
| Shannon`s diversity indicator | SHDI | =$-\sum_{i=1}^{m} (p_{i}*lnp_{i})$ | SHDI is a measure of patch classes diversity in landscape (Nagendra, 2002),$p_{i}$ is the proportion of the landscape occupied by patch class i.  SHDI=0 when the landscape is no diversity. Low values of SHDI represent low landscape diversity, and high values of SHDI represent high landscape diversity. |
| Patch richness indicator | PR | =m | PR is the meaning of the number of patch classes (Baldwin et al., 2004), m is the number of different landscape patch classes.  PR=1 when the landscape contains only 1 path class. Low values of PR represent low richness of patch classes, and high values of PR represent high richness of patch classes. |
| Number of patches indicator | NP | =$\sum_{i=1}^{m} {np}_{i}$ | NP is the total number of patches in the landscape(Cain et al., 1997), ${np}_{i}$ is the number of patches within path class i.  NP=1 when the landscape contains 1 patch. Low values of NP represent low number of patches, and high values of NP represent high number of patches. |

**TABLE S2** Calculation formulae of the FLIs

| Names | Codes | Formulae | Comments |
| --- | --- | --- | --- |
| Fusion Shannon’s diversity indicator | FLI-SHDI | =$-\sum_{i=1}^{m} (p_{i}*lnp_{i}*(1-(p_{i}*\sum_{j=1}^{n} \left( p_{ij}*\ln\left( p_{ij} \right) \right)/\ln\left( n_{i} \right)))$ | $p_{i}$ is the proportion of the landscape occupied by patch class i; $p_{ij}$ is the proportion of the second-grade level patch class j in the first-grade level patch class i ; and $n_{i}$ is the number of second-grade level landscape classes occupied by the first-grade level. |
| Fusion path richness indicator | FLI-PR | =$\sum_{i=1}^{m} ({pr}_{i}*(1-(p_{i}*\sum_{j=1}^{n} \left( p_{ij}*\ln\left( p_{ij} \right) \right)/\ln\left( n_{i} \right)))$ | If the landscape region contains first-grade level patch class i; then ${pr}_{i}$ equals 1; else ${pr}_{i}$ equals 0. |
| Fusion number of patches indicator | FLI-NP | =$\sum_{i=1}^{m} ({np}_{i}*(1-(p_{i}*\sum_{j=1}^{n} \left( p_{ij}*\ln\left( p_{ij} \right) \right)/\ln\left( n_{i} \right)))$ | ${np}_{i}$ is the number of patches of the first-grade level path class i. |

FLI-SHDI is a Shannon’s diversity indicator based on two-grade patch classes, whose value represents the amount of information entropy per G1 patch class and G2 path class. FLI-PR measures the richness or richness density of two-grade patch classes, which are not affected by the spatial arrangement of landscape patterns. And FLI-NP measures the number of patches or patch density, which fuse G1 and G2 patch classes.

**TABLE** **S3** The classification table of LUCC dataset

| G1 (First Grade of LUCC) | | G2（Second Grade of LUCC） | |
| --- | --- | --- | --- |
| G1 categories codes | G1 classes | G2 categories codes | G2 classes |
| 1 | Agricultural land | 11 | Paddy field |
|  |  | 12 | Dry farm |
| 2 | Forestland | 21 | Woodland |
|  |  | 22 | Spinney |
|  |  | 23 | Open woodland |
|  |  | 24 | Other woodland |
| 3 | Meadowland | 31 | High-coverage grassland |
|  |  | 32 | Middle-coverage grassland |
|  |  | 33 | Low-coverage grassland |
| 4 | Wetland | 41 | Rivers and canals |
|  |  | 42 | Lakes |
|  |  | 43 | Reservoir pit pond |
|  |  | 44 | Swampland |
|  |  | 45 | River rapids |
| 5 | Constructive land | 51 | Urban land |
|  |  | 52 | Rural residences |
|  |  | 53 | Other buildings |
| 6 | Unutilized land | 61 | Desert |
|  |  | 62 | Gobi land |
|  |  | 63 | Saline-alkali land |
|  |  | 64 | Glacier |
|  |  | 65 | Bare land |
|  |  | 66 | Rocky soil |
|  |  | 67 | Other unutilized land |

**TABLE S4** The relationship between the LPIs and their corresponding information volumes across 3 indicators, using the spatial matching Pearson correlation coefficient method

| Types | SHDI | PR | NP |
| --- | --- | --- | --- |
| FLIs, Information volume of FLIs | -0.19^*^ | -0.34^**^ | 0.26^**^ |
| G1LIs, Information volume of G1LIs | -0.10 | -0.39^**^ | 0.23^*^ |
| G2LIs, Information volume of G2LIs | -0.27^*^ | -0.08 | 0.32^**^ |

Notes: ^*^ means correlation significant at the 0.05 level, ^**^ means correlation significant at the 0.01 level.

**TABLE S5** The multiple linear regression equations of information volume for SHDI, PR, and NP

| Types | Multiple linear regression equations | R^2^ | F value | P value |
| --- | --- | --- | --- | --- |
| SHDI | \| Y= -0.11+0.75×X_1_+0.27×X_2_ \| \| --- \| | 0.92 | 5724.01 | <0.001 |
| PR | \| Y= 4.32+0.71×X_1_+0.23×X_2_ \| \| --- \| | 0.55 | 600.97 | <0.001 |
| NP | \| Y= 1.01+0.90×X_1_+0.14×X_2_ \| \| --- \| | 0.94 | 8102.19 | <0.001 |

Notes: The confidence level is set to 99%, Y is the information volume of FLIs, X_1_ is the information volume of G1LIs, and X_2_ is the information volume of G2LIs

FIGURE


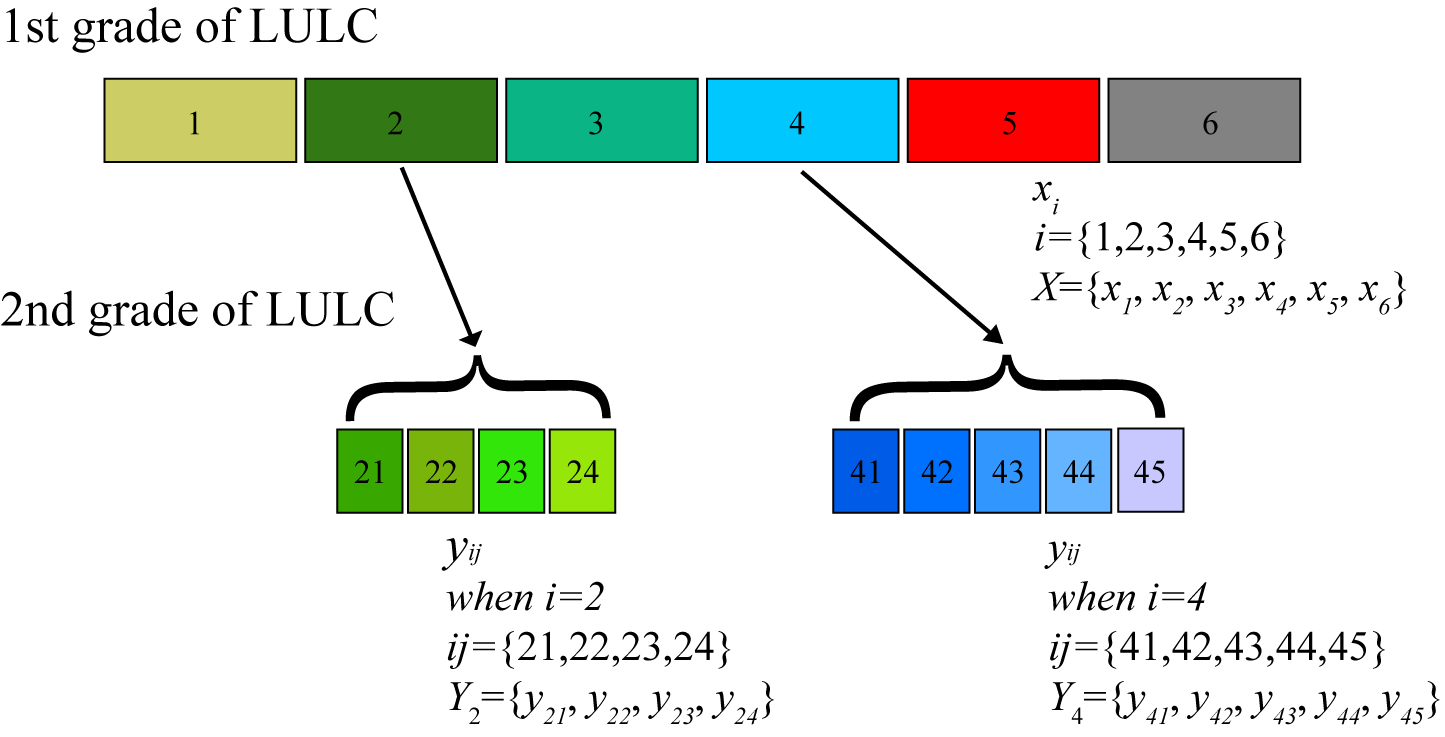


**FIGURE S1** An example of the relationships between x_i_, Y_i_, and y_ij_ based on land use and land cover (LULC)

FIGURE S1 shows the relationships between x_i_, Y_i_, and y_ij_ of two-grade LULC in manuscript. We take it as an example to illustrate how the equations work. If the X set with area proportion array P= {0.1, 0.3, 0.2, 0.2, 0.1, 0.1}, and the Y_2_ set with total area proportion array P_2_= {0.01, 0.03, 0.04, 0.02}. Then $H\left( X \right)=-\sum_{x_{i}X} p\left( x_{i} \right)*l_{n}p\left( x_{i} \right)$=1.696, $p\left( y_{21} | x_{2} \right)=p(y_{21})/p(x_{2})$=0.01/0.1=0.1. Therefor $H_{2}^{’}=-p(x_{i})*\sum_{y_{ij} Y_{i}} p\left( y_{ij} | x_{i} \right)* ln\left( p\left( y_{ij} | x_{i} \right) \right)$=$-p\left( x_{2} \right)*\sum_{y_{2j} Y_{i}} p\left( y_{2j} | x_{2} \right)* ln\left( p\left( y_{2j} | x_{2} \right) \right)$= 0.128, and $w_{2}=H_{2}^{’}/ln\left( n_{2} \right)$=0.128/ln(4)=0.092. And the more details have been provided on <https://github.com/ecofg/FLIs_fusion-metrics>. By using the Python codes, the two-grade land use data can be calculated to obtain the FLIs.





**FIGURE S2** The spatial Pearson correlation coefficient heat map





**FIGURE S3** The total information volume of the FLIs and LIPs on a series of moving window scales


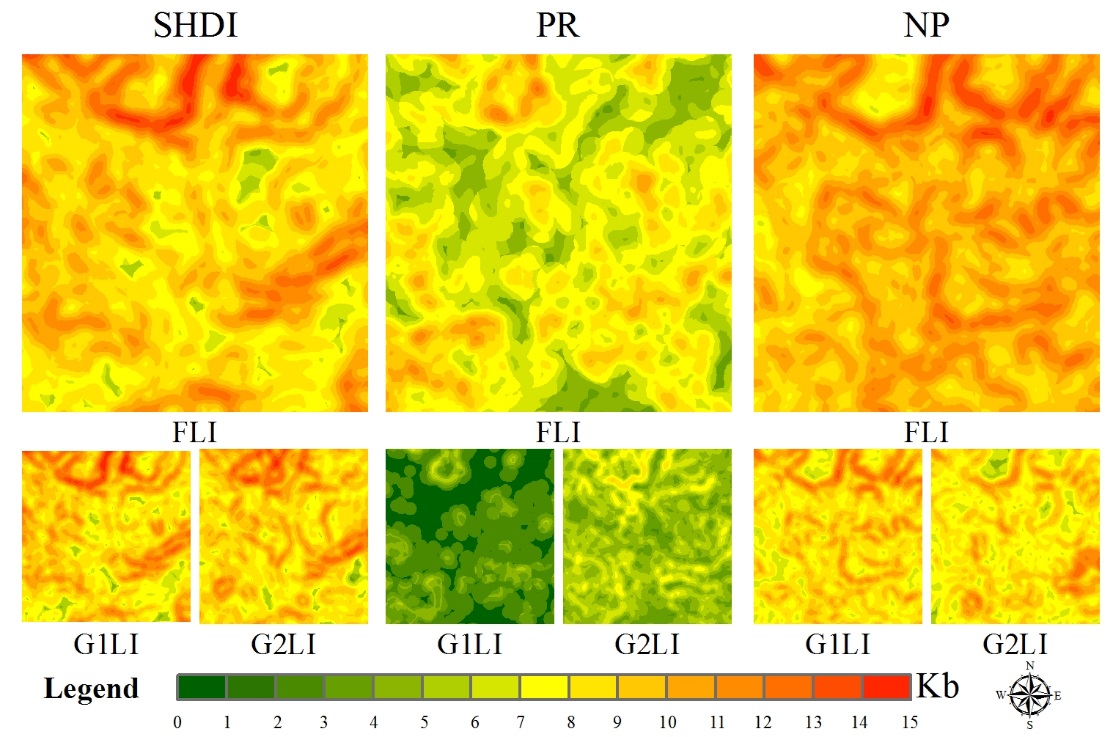


**FIGURE S4** The spatial distributions of information volume of the FLIs, G1LIs and G2LIs / KB, KB is 2^10^ bits

**Information volume**

The Huffman optimal coding method for computing minimum-redundancy has been widely used in data compression (Chung, 1997; Moffat, 2019). And it is one of the entropy encoding approaches for the compression of data (Khaitu and Panday, 2018). Most of the redundancy of the data can be eliminated by the Huffman coding’s minimum-redundancy compression, so the compressed data can reflect the information volume contained in the data. And the Huffman tree is also called the optimal binary tree, which has the shortest weighted path length (Chung, 1997; Moffat, 2019).

We take an example to show the structure of Huffman tree. An array of symbols S= {A, B, C, D, E, F, G} with frequencies array W= {2,1,3,4,5,1,7}, respectively. Usually, in the binary alphabet, each letter uses 8 bits, such as the binary character of the letter A is “01000001”. So the total information volume of example data is 184 bytes without any compression. According to the Huffman optimal coding method, the Huffman tree is demonstrated in Figure S5. The weighted path length of the Huffman tree (*WPL*) is the sum of the weighted path lengths of all leaf nodes in the tree, and the *WLP* is calculated by

*WPL*=$\sum_{i=1}^{n} w_{i}*l_{i}$ （S1）

where *n* is the number of leaf nodes, $w_{i}$ is the weight of the leaf node (e.g., the frequency), and $l_{i}$ is the length of the path from the root node to the leaf node (e.g., 1 bit).


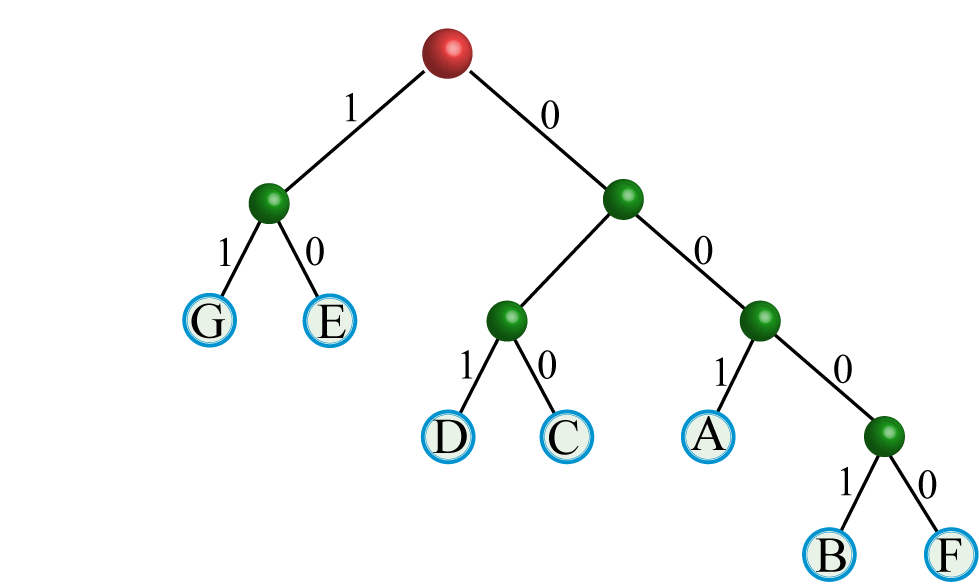


**FIGRE S5** Huffman binary tree of example data

Considering this Huffman binary tree (Figure S5), the letter A is compressed as “001” only containing 3 bits. Using the *WPL* as the sum binary bytes of all letters, the new information volume of data is 59 bits, which is compressed by 67.9%.

Because the dimension and unit of the landscape pattern indicators (LPIs) are inconsistent, it may cause serious system bias in comparing their information volume. Therefore, in order to accurately compare the information volume of LPIs, we respectively normalize the values of LPIs, with a value range of [0,1]. The different number of decimal places of LPIs’ normalized values will lead to a great difference in the information volume. For example, a normalized value is 0.2536329, if we respectively round it to 2 decimal places and 4 decimal places of it, the values are 0.25 and 0.2536. As the number of decimal places increases, the value becomes more accurate, but the importance of the lower numerical value is reduced, such as the hundredth place of a decimal is more important than the ten thousandth place. There are two methods in the information volume statistics. Among them, the simple calculation method is to uniformly round the same decimal places for all normalized values (e.g., rounding them to 4 decimal places) and then calculate their information volume using formula (S1). Meanwhile, another more accurate method is to calculate their information volume from one decimal place to *m* decimal places in turn, and then get their sum volume based on their weight, that is

$SIV=\sum_{j=1}^{m} w_{j}*{WPL}_{j}$ (S2)

Where *SIV* is the sum of information volume, *j* is the number of decimal places reserved for normalized values, $w_{j}$ is the weight of ${WPL}_{j}$.

In this study, we set $w_{j}={{0.5}^{j}}/{{(1-0.5}^{m})}$ and *m*=4. What needs to be emphasized is that when *m*=4, the *SIV* is greater than 90% of the actual total information volume, so there is no need to calculate all significant digits of normalized values. Meanwhile, it should note that if the simple calculation method of information volume is adopted, the *WPL* with rounding to 4 decimal places of values also can be regarded as the information volume instead of the *SIV*. And the Python codes and more details have been provided on <https://github.com/ecofg/FLIs_fusion-metrics>.

**REFERENCES**

Baldwin, D.J.B., Weaver, K., Schnekenburger, F., & Perera, A.H. (2004). Sensitivity of landscape pattern indices to input data characteristics on real landscapes: Implications for their use in natural disturbance emulation. *Landscape Ecology*, 19, 255-271. https://doi:10.1023/B:LAND.00000 30442.96122.ef.

Cain, D.H., Riitters, K., & Orvis, K. (1997). A multi-scale analysis of landscape statistics. *Landscape Ecology*, 12, 199-212. https://doi:10.1023/A:1007938619068.

Chung, K.L. (1997). Efficient Huffman decoding. *Information Process Letter*, 61, 97-99. https://doi:10.1 016/S0020-0190(96)00204-9.

Khaitu, S.R., & Panday, S.P. (2018). Canonical huffman coding for image compression, *in*, eds. S.D. Sudarsan, V. Kumar & R. Tomar., 184-190.

Moffat, A. (2019). Huffman coding. ACM Comput. Surv. 52. https://doi:10.1145/3342555.

Nagendra, H. (2002). Opposite trends in response for the Shannon and Simpson indices of landscape diversity. *Applied Geography*, 22, 175-186. https://doi:https://doi.org/10.1016/S0143-6228(02)00002-4.
