# Supplementary figures and images for: Fusing multidimensional hierarchical information into finer spatial landscape metrics
Source: Ecol Evol. 2021 Oct 12;11(21):15225–36. doi: 10.1002/ece3.8206 (PMC8571621; doi:10.1002/ece3.8206)

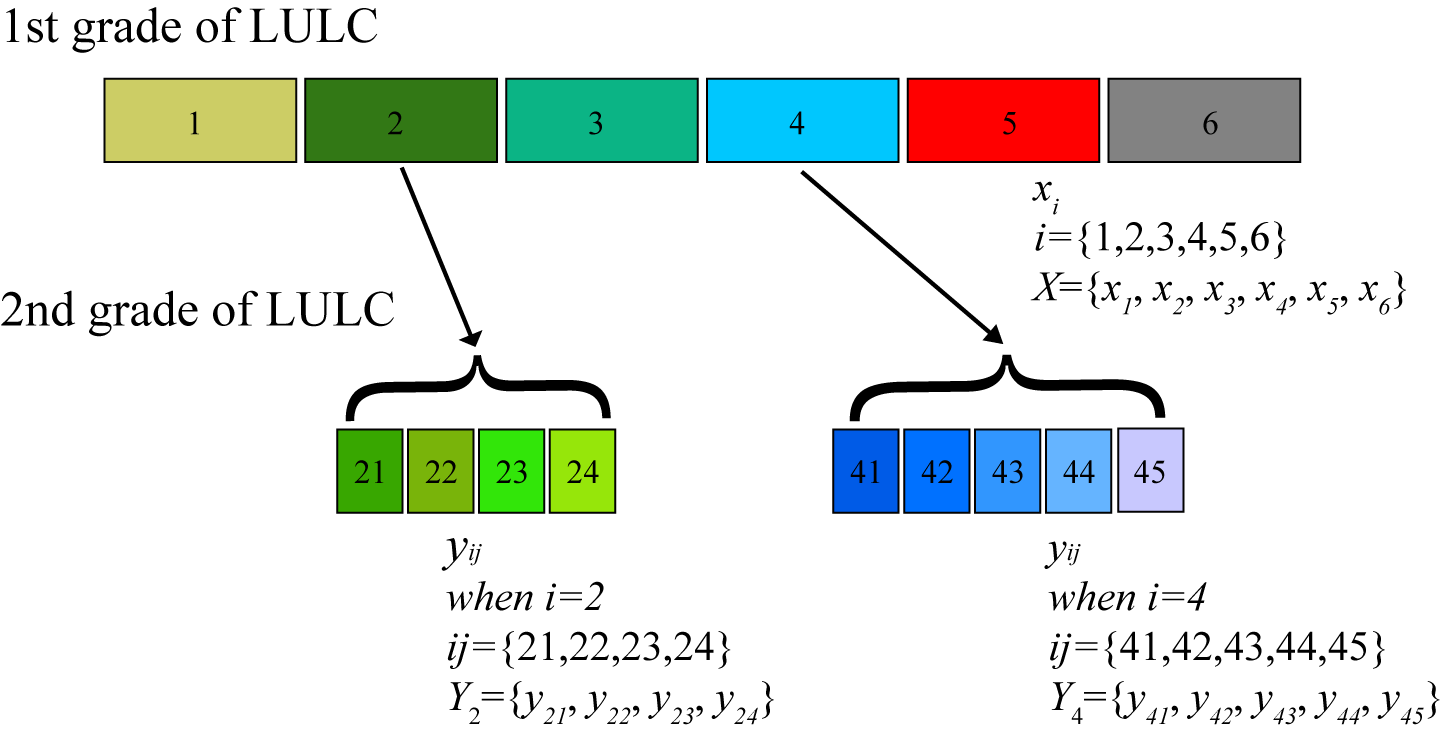

Supplement: Supplementary file 2 — Fig S1 [file ECE3-11-15225-s001.tif]

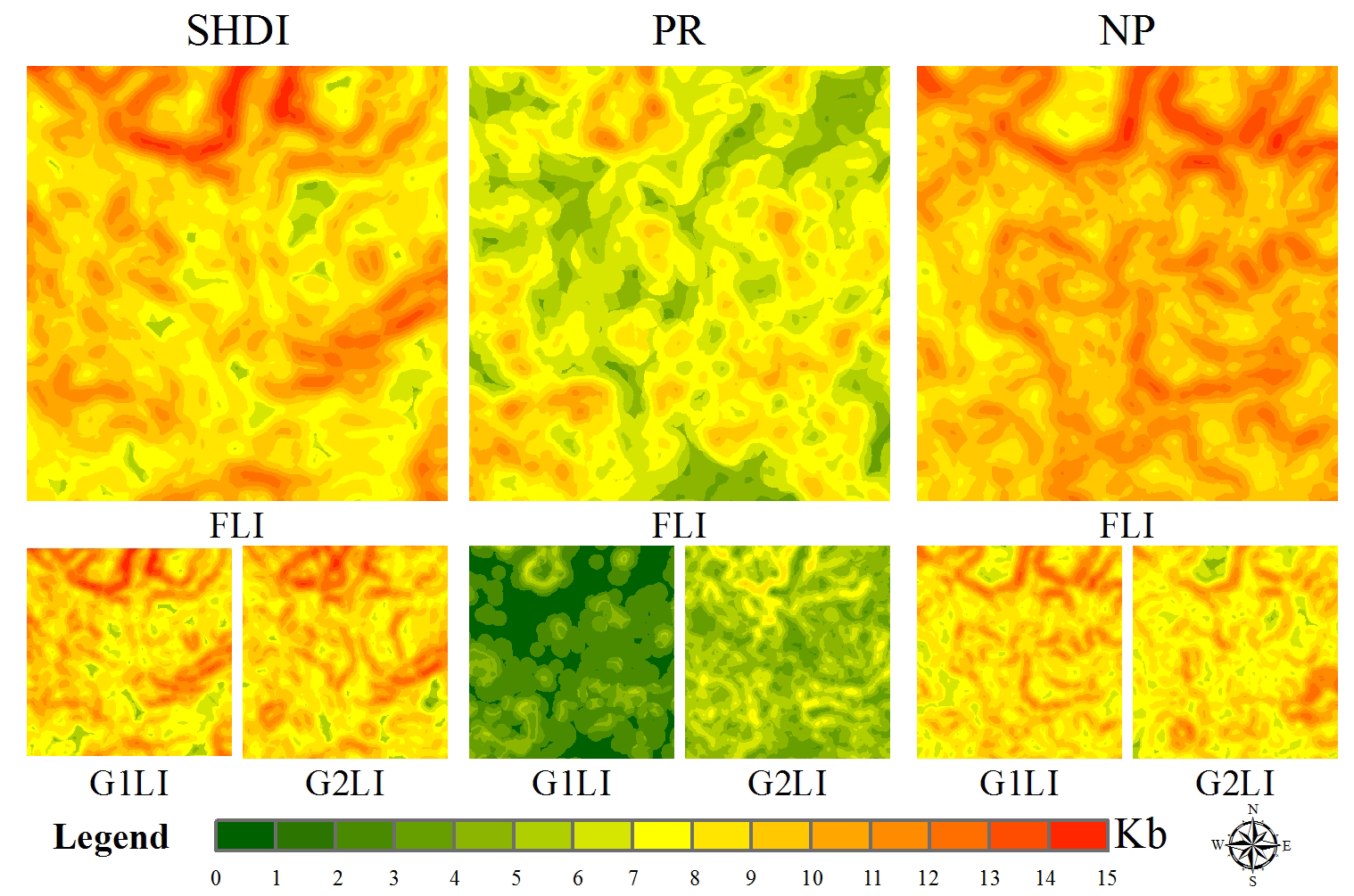

Supplement: Supplementary file 5 — Fig S4 [file ECE3-11-15225-s004.tif]

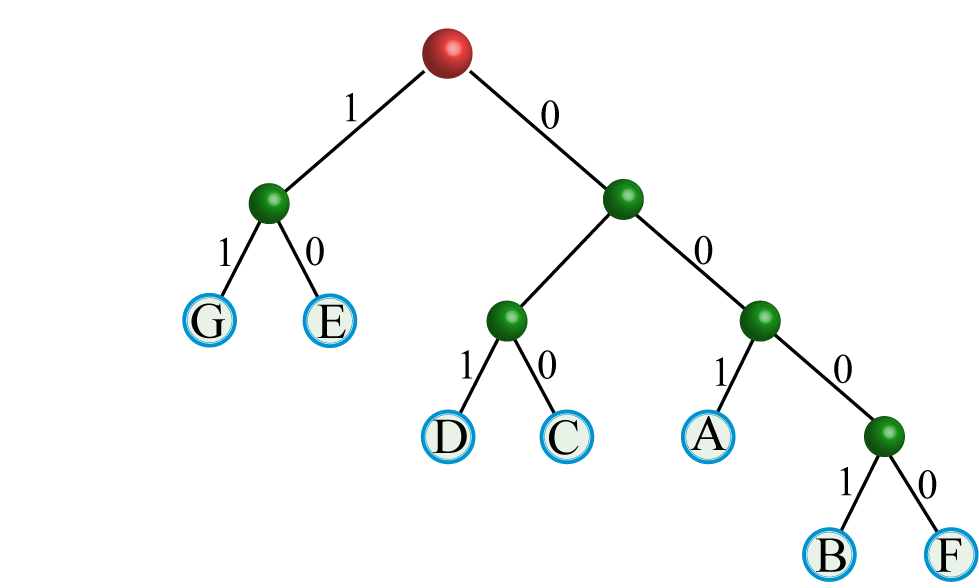

Supplement: Supplementary file 6 — Fig S5 [file ECE3-11-15225-s002.tif]
